# Annotations

**Published:** 1898-06-25

**Authors:** 


### Annotations.

### Hospitals and Accidents.

One of the very first duties of a hospital is to be ready at all times to receive all sorts of cases of accident or emergency. This seems so obvious a platitude that we should not have thought of repeating it were it not one of the very points that some hospital authorities seem most prone to forget. We have lately heard of a case in which disastrous results followed the refusal to take charge of a case of emergency, notwithstanding that the refusal was apparently based on a perfectly proper desire to save the patient from running the risk of catching an infectious disease. The case to which we refer is that of a man who recently died in the Manchester Infirmary. It appears that on the morning of May 13th he sustained what is described as a compound fracture of the right knee-cap, and after being attended to temporarily by a medical man he was sent on an ambulance to the Victoria Hospital, Burnley. For some reason or other, apparently because there had been scarlet fever in the hospital, it was thought best to remove him the next day to the workhouse infirmary. Unfortunately, however, the man having passed a night in the hospital and having thus been exposed to the infection of scarlet fever, he was refused admission on arriving at the workhouse infirmary, and so had to be taken home, where he arrived on the evening of the day after the accident. After another two or three days, he was carried off to the Manchester Royal Infirmary, where he was admitted, and where he ultimately died. We do not wish to attribute blame to any individual. The jury who investigated the circumstances of the case returned a verdict of "Accidental death," and behind this verdict we do not wish to go. What, however, we do think it right to point out is that a hospital should so organise its resources and arrange its work as to be ready at any time to receive the accidents and emergencies which may be brought to it. When a hospital is worked up to the full capacity of its beds it is neglecting one of the prime functions for which it was built, namely, to be an ever-ready refuge in case of accident; while, if a hospital is furnished with so poor a supply of isolation wards that the presence of a case or two of scarlet fever renders it a place to which accidents cannot be safely taken, we cannot look upon it as in a satisfactory condition. Seeing no hospital can protect itself from infectious cases, and seeing the frequency with which they are apt to occur in any general hospital, however great may be the care taken to guard against their admission, every hospital should be prepared to deal with with infectious cases as they arise.

### Paris and London Water.

From the evidence given by M. Gaston Cadoux, the Chef de Bureau at the Prefecture of the Seine, before the Royal Commission on London Water Supply, it would appear that a very interesting bacteriological experiment is periodically tried in Paris on a very large scale. People in Paris have a double water supply, spring water used for drinking and domestic purposes, and Seine water for municipal purposes. The pipes are quite separate and distinct. Unfortunately, however, the spring water is apt to run short, and when this happens they have to pump Seine water into the spring water reservoirs. "They have to pump up the bad water, if I may use that term, into the reservoir, which is generally full of pure, good water," and send it along the pipes that should have pure water in them. Now, when this is done the pipes become infected with the bacteria carried by the Seine water. The result is curious. When they turn spring water into the pipes that have been carrying river water an enormous development of bacteria occurs. Then, after the pure water has gone through the pipes for two or three days, the development ceases, all of which is in accordance with well-known bacteriological experience. Perhaps it is something of the same sort that happens now and again when London water is found temporarily to contain large numbers of microbes. Not that the London water companies turn their water into dirty pipes. But if an "infection" of pure water by dirty pipes will produce such an enormous development of microbes, an "infection" by the passage of a little unfiltered or ill-filtered river water would have the same effect. When great numbers of microbes are found in London water, as does happen now and again, it is not necessary to imagine that all these have passed the filters. Only a very small number may have got through, but sufficient to inoculate the mass, just as the trace remaining in the pipes of Paris infects the pure water flowing through them. It is of small use to have pure water as a general thing, if it is left subject to occasional pollution even by comparatively small quantities of unfiltered river water; for, small as it may be, the microbes it contains will, as M. Cadoux says, "multiply enormously" in the pure water and thus defile it.

### The Health of Liverpool.

In the recently-issued report on the health of Liverpool during the year 1897 by Dr. Hope, medical officer of health, there are two points which are of special importance. One is the proof that is given that the method adopted by the Registrar-General for estimating the population leads to erroneous results. That a moderate degree of error should creep in is no doubt inevitable, but that in a city like Liverpool an error of 100,000 should have arisen towards the close of the last inter-censal period is a matter of much concern. For a city whose population is over-estimated will show a fictitious decline in its rate of sickness and mortality, perhaps leading the sanitary authority to refrain from necessary expenditure, while on the other an under-estimate gives rise to a death-rate higher than is correct, and useful measures which are doing good work may thus be discredited and abandoned. The cure for this state of affairs would appear to be a more frequent census. The other point which we think is of especial interest is the demonstration given by Dr. Hope of the evils resulting from the unrestricted building of back-to-back houses. The plans which he publishes show a state of affairs which never ought to have been allowed to grow up, and amply prove the urgent necessity for well-considered building laws in every town. Taking the whole of certain insanitary areas the average death-rate was 42 per 1,000, but varied in different groups of houses, running up in the Paul Street group to the frightful mortality of 79·6 per 1,000!

